# Rumen Inoculum Collected from Cows at Slaughter or from a Continuous Fermenter and Preserved in Warm, Refrigerated, Chilled or Freeze-Dried Environments for In Vitro Tests

**DOI:** 10.3390/ani9100815

**Published:** 2019-10-16

**Authors:** Mauro Spanghero, Maria Chiaravalli, Stefania Colombini, Carla Fabro, Federico Froldi, Federico Mason, Maurizio Moschini, Chiara Sarnataro, Stefano Schiavon, Franco Tagliapietra

**Affiliations:** 1 Dipartimento di Scienze Agroalimentari, Ambientali e Animali, University of Udine, 33100 Udine, Italy; carla.fabro@uniud.it (C.F.); sarnataro.chiara@spes.uniud.it (C.S.); 2 Dipartimento di Scienze Agrarie e Ambientali, University of Milan, 20122 Milano, Italy; maria.chiaravalli@unimi.it (M.C.); stefania.colombini@unimi.it (S.C.); 3 Dipartimento di Scienze Animali, della Nutrizione e degli Alimenti, Università Cattolica del Sacro Cuore of Piacenza, 29122 Piacenza, Italy; federico.froldi@unicatt.it (F.F.); maurizio.moschini@unicatt.it (M.M.); 4 Department of Biodiversity Protection, Institute of Animal Reproduction and Food Research of Polish Academy of Sciences (IARFR PAS), 10-748 Olsztyn, Poland; f.mason@pan.olsztyn.pl; 5 Dipartimento di Agronomia, Animali, Alimenti, Risorse naturali e Ambiente, University of Padova, 35122 Padova, Italy; stefano.schiavon@unipd.it (S.S.); franco.tagliapietra@unipd.it (F.T.)

**Keywords:** rumen liquid, in vitro fermentation, rumen degradability, gas production

## Abstract

**Simple Summary:**

The utilization of animal donors of rumen fluid for laboratory experiments can raise ethical concerns due to invasive methods of collection (rumen cannulated or intubated animals). Societies are strongly oriented to support cruelty free experiments and alternatives to the collection of rumen fluids from live animals are urgently requested from the scientific community. Thus, in order to attenuate the dependence of laboratories on animal donors, this study compared the rumen inoculum collected at slaughter with the fermentation liquid from a rumen continuous fermenter and both rumen inoculum were used fresh or preserved (by refrigeration, chilling and freeze-drying). The results support the possibility of using continuous fermenters to generate inoculum for in vitro purposes, and short-term refrigeration is confirmed to be a valuable storage system to facilitate transfer inoculum from the collection sites. These findings should attenuate the need for laboratories’ frequent collections from animals while continuing research in ruminant nutrition.

**Abstract:**

The utilization of animal donors of rumen fluid for laboratory experiments can raise ethical concerns, and alternatives to the collection of rumen fluids from live animals are urgently requested. The aim of this study was to compare the fresh rumen fluid (collected at slaughter, W) with that obtained from a continuous fermenter (RCF) and three methods of rumen fluid preservation (refrigeration, R, chilling, C, and freeze-drying, FD). The fermentability of different inoculum was evaluated by three in vitro tests (neutral detergent fiber (NDF) and crude protein (CP) degradability and gas production, NDFd, RDP and GP, respectively) using six feeds as substrates. Despite the two types of inoculum differed in terms of metabolites and microbiota concentration, the differences in vitro fermentability between the two liquids were less pronounced than expected (−15 and 20% for NDFd and GP when the liquid of fermenter was used and no differences for RDP). Within each in vitro test, the data obtained from rumen and from fermenter liquids were highly correlated for the six feeds, as well as between W and R (r: 0.837–0.985; *p* < 0.01). The low fermentative capacity was found for C and, particularly, FD for liquids. RCF could be used to generate inoculum for in vitro purposes and short-term refrigeration is a valuable practice to manage inoculum.

## 1. Introduction

Rumen fluid sampled from live animals is used in laboratory experiments (e.g., in vitro rumen fermentation) to evaluate the nutritive value and gaseous emissions of ruminant feeds [1,2] or to inoculate continuous fermenters for studies of rumen fermentation [3].

In general, the utilization of animals as donors of rumen fluid can raise some ethical concerns, because the collection of rumen fluid is an invasive practice, which requires donor animals that are surgically modified (e.g., rumen cannulated), or immobilised and intubated with esophageal probes. An alternative is to collect rumen fluid from animals slaughtered for production purposes in commercial slaughterhouses, but it is difficult to monitor feeding before slaughter. Overall, societies are strongly oriented to support cruelty free experiments and alternatives to collection of rumen fluids from live animals are urgently requested of the scientific community to continue research activity in ruminant nutrition.

Rumen continuous fermenters (RCFs) are laboratory apparatus developed to simulate the rumen conditions for studies of rumen metabolism. They generate a fermenting fluid by starting from an initial rumen inoculum and by a continuous influx of artificial saliva, an output of fermentation products and a constant supply of nutrients (substrates). However, they could also be modified and adapted to be used as artificial generators of rumen fermentation fluid, which could be standardised with respect to several conditions (type and amount of fermentable substrate, pH, dilution, etc.). There are studies which have compared fermentation liquids from different RCFs or between fluids collected from rumens and fermenters [3,4,5,6,7]. The liquid from fermenters is less concentrated in terms of volatile fatty acids (VFA) and protozoa, and the cellulosolitic bacterial strains seem reduced in some types of RCF (e.g., Rusitec, [8]) while in other fermenters, the bacteria microbiota was comparable to that measured directly on the rumen inoculum collected in vivo [6,7]. However, no experiments have simultaneously compared rumen and RCFs fluids as inoculum in terms of results of different in vitro tests.

Overall, independently from the mode of inoculum provision, the possibility of preserving rumen fluid and to create stocks would allow the concentration of the collection in specialized centers to facilitate the transfer of the inoculum and reduce the need of laboratories to frequently collect liquids from live animals. The preservation techniques of rumen fluid have been investigated in several studies, which have mainly considered the usage of low temperatures (e.g., refrigeration and chilling at −20, at −80 °C, or in liquid N), the addition of cryoprotectants, and also freeze-drying [9,10,11,12,13]. In general, there are encouraging findings, but also a scarce homogeneity among trials in terms of measurement of the maintenance of the fermentative capacity of liquid after preservation.

The present research has the general aim to attenuate the needs of direct collection of rumen fluid from animals by: (i) artificially generating rumen fermenting fluids; (ii) evaluating preservation methods for stock rumen fluid. These technologies should reduce the need for laboratories to frequently collect from animals, with the potential advantage of reducing inoculum variability. The specific aim is to compare the fresh rumen fluid (collected at slaughter) with that obtained from a stratified single-flow RCF system [14] and three methods of rumen fermenting fluid preservation (refrigeration, chilling and freeze-drying).

Unlike other research, the novelty of this study is to compare the different inoculum in terms of the results obtained by three widely utilised in vitro methods (degradability of neutral detergent fiber, NDFd, degradability of protein (RDP), and gas production, (GP) respectively). The chosen in vitro tests allow a wide evaluation of the fermentation potential of inoculum, because they quantify the fermentation of main dietary components of ruminant rations, such as fibers (NDFd), protein (RDP) and (mainly) non-fibrous carbohydrates (GP).

## 2. Materials and Methods

### 2.1. Trial Organization

The experiment was organised in two subsequent identical fermentation trials (runs) and was carried out by four Italian research groups from the University of Milano, Padova, Piacenza, and Udine (Labs 1, 2, 3, and 4, respectively).

**Figure 1:**
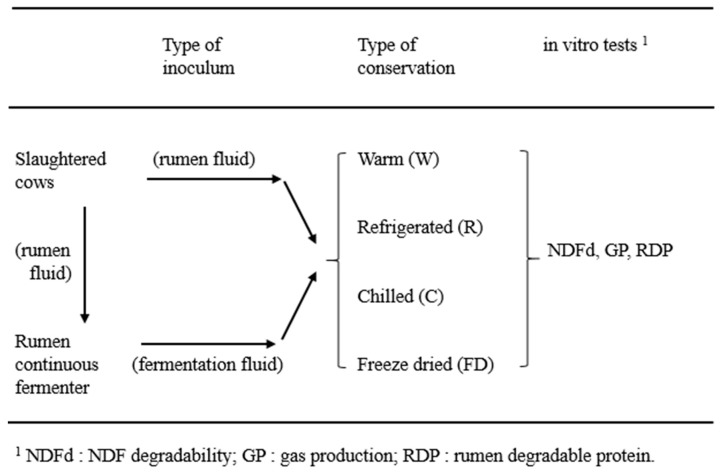
Schematic representation of the trial organization.

For each trial, 4 dry multiparous Holstein Friesian cows were slaughtered for production purposes in a commercial slaughterhouse, after being fed for 3 weeks a basal diet (32.3% meadow hay, 34.0% corn silage, 27.3 % compound feed and 6.4 % soybean meal). The cows of both runs were in good health, housed in the same barn located 50 km from the slaughterhouse, fed the last feeding in the morning 2 h before being moved to the slaughterhouse, and had free access to fresh water until slaughter. After slaughter, the rumen liquors, collected in equal amounts from the 4 cows by Lab 4, were coarsely filtered, the liquids bulked together during continuous flushing with CO_2_, and then divided into 2 main amounts (see Figure 1). The first amount represented the rumen inoculum for the in vitro tests (NDFd, RDP and GP test), while the second amount was used to inoculate the RCF system. Both inoculum were used as: warm at 39 °C (W), refrigerated at 4 °C (R), chilled at −80 °C (C), and freeze-dried (FD). After the collection at slaughter, W (kept inside pre-warmed thermic bottles flushed with CO_2_) and R (kept inside bottles flushed with CO_2_, immersed in ice water within a portable fridge to quickly lower the temperature to 4 °C) were divided in aliquots of 250–300 mL each and immediately delivered to Lab 1, 2, and 3 to start the in vitro tests within 6 h from slaughter. The amounts to be preserved as C, FD or to be used in the RCF fermenter were immediately brought to Lab 4 (maintained warm at 39 °C). Here, the C or FD, amounts were divided in aliquots of 250–300 mL each. The C aliquots were chilled at −80 °C, while the FD aliquots were processed according to Luchini et al. [15]. Briefly, after centrifugation at 5000 g for 30 min at 4 °C, the supernatant was discarded, and the residue obtained was chilled at −80 °C, and subsequently freeze-dried. The C and FD aliquots were delivered to Lab 1, 2, 3 after 30 d of conservation. Before being used as inoculum, the C liquids were thawed in a bain-marie at 39 °C within 2 h and kept at the same temperature for another 2 h, while the FD liquids were reconstituted to the original volume with the artificial saliva used for the in vitro tests and kept at 39 °C for 2 h.

The remaining amount of rumen liquid was immediately used to inoculate the RCF fermenter in Lab 4. On the 9th day of incubation in the RCF, the fermentation fluid was collected, and divided in aliquots for each liquid type (W, R, C and FD). The W and R aliquots, prepared as previously described for the rumen inoculum, were immediately transferred to Lab 1, 2 and 3 to perform the in vitro tests within 6 h from the collection of the liquid from the RCF system. The C and FD aliquots were delivered to Labs 1, 2, and 3 after 30 d of conservation.

As incubations of liquids C and FD were delayed by 30 d with respect to W and R and the incubations of liquid from the fermenter were delayed by 9 d from the incubation of rumen liquids. There were in total 4 incubation sessions within each fermentation run.

### 2.2. Preparation of the Substrates for the In Vitro Tests

All the in vitro tests were performed on the following six ingredients: meadow hay, corn silage, wheat bran, distillers, soybean and barley meal. The samples of corn silage were dried (48 h at 60 °C) and all feed samples were milled through a 1 mm sieve and then analysed for chemical composition.

### 2.3. The Rumen Continuous Fermenter (RCF) System and the In Vitro Tests

The RCF system, used in Lab 4, was described in detail by Mason et al. [14]. In brief, the system consists of 8 × 2 L glass bottles, immersed in a water bath at 39 °C. A peristaltic pump supplies the buffer solution [16] from a reservoir to the fermenters (dilution rate of 5 %/h) and the outflow, located at the bottom of the bottles, allows stratification of the feeding material. The bottles were inoculated with 600 mL of strained rumen fluid and 800 mL of artificial saliva, and each bottle received a total of 15 g/d of dry matter (DM), in two equal doses, at 09:00–17:00, of the same diet used to feed the donor cows before slaughtering. Each fermentation lasted 9 d and on the last day, the fermentation fluids of the 8 bottles were collected, pooled, and processed to prepare the different inoculum, as previously described.

In all 3 in vitro tests, the inoculum was strained through 4 layers of cheesecloth into pre-warmed (39 °C) flasks with CO_2_ and mixed with the buffer solutions ([17] for NDFd and GP and Van Soest buffer [18] for RDP, in a 1:2 and 1:4 ratio, respectively).

The NDFd was tested by Lab 1. Each feed sample was weighed (0.250 ± 0.005 g) in duplicate in Ankom F57 bags (Ankom Technology, Macedon, NY, USA). The bags were incubated in a pre-warmed 100 mL Erlenmeyer flask closed by a rubber stopper with a Bunsen valve for gas release and maintained at 39 °C in a water bath with shaking for 48 h. Each flask was inoculated with 90 mL of the solution under anaerobic conditions, flushing the flask with CO_2_. At the end of the incubations, the bags were rinsed with cold water until the water ran clear and then placed for 3 h in a 105 °C forced-air oven to dry. Subsequently, the NDF concentration was determined for each bag using the fiber analyser (Ankom Technology, Macedon, NY, USA).

The RDP was tested by Lab 3 according to the rumen step of the Ross method [19]. Briefly, each sample was weighed (1.000 ± 0.020 g) into a 120 mL glass bottle equipped with a cap and placed into a 39 °C water bath 1 h before the in vitro fermentation. The neutral detergent residue from corn silage was used for microbial contamination correction of the post fermentation feed residues. Each bottle was inoculated with 100 mL solution under CO_2_ flushing, closed with the cap, and incubated for 16 h at 39 °C in a shaking water bath. After the incubation, the bottle content was vacuum filtered (110 mm diameter Whatman Filter Papers 54) and the residue was analyzed for Kjeldahl nitrogen.

The GP was measured after 24 h incubation by Lab 2 by using a commercial GP apparatus (Ankom Technology, Macedon, NY, USA; [20]). The system consists of 50 bottles hermetically sealed, equipped with a wireless pressure sensor connected to a computer. Each bottle (317 mL) was filled with 0.500 ± 0.010 g of feed and 75 mL of fermenting solution, obtaining a headspace volume of 242 mL. The bottles were placed in a ventilated incubator at 39 ± 0.4 °C and automatically vented at a fixed pressure (6.8 kPa), to prevent overpressure.

All the tests were performed for each feed substrate and for each inoculum type in duplicate (8 inoculum types × 6 feeds in duplicate) and were replicated in a second fermentation run. The between-run determinations were considered as experimental repetitions. To account for the incubation session effect, a standard rumen fluid was included in each incubation session by each laboratory. Before conducting the whole experiment, Lab 4 prepared a rumen fluid to be used as the control by other labs. The liquid was collected at slaughter from 4 dairy cows (culled in good health) and delivered to Lab 4 within 30 min in airtight glass-bottles refluxed with carbon dioxide and maintained at 39 °C. The whole amount was divided in small aliquots (200 mL), frozen at −20 °C and distributed to Lab 1, 2 and 3. The frozen-thawed inoculum (in a bain-marie at 39 °C within 2 h and kept at the same temperature for another 2 h) were added by each Lab into each incubation run (two incubation bottles added with corn silage as substrate) to detect anomalies between runs.

### 2.4. Analysis

#### 2.4.1. Inoculum Sample Preparation

The samples of the two inoculum for each preservation treatment were collected before performing the in vitro tests for the following analyses: pH, VFA, NH_3_, and microbial population. The samples for the VFA analysis were acidified with H_2_SO_4_ 0.1 N and stored at −20 °C while the samples for NH_3_ were directly stored at −20 °C. The samples for bacterial DNA analysis were chilled in liquid nitrogen (N) and stored at −80 °C.

#### 2.4.2. Chemical Analysis

The DM content of the feeds was determined by heating at 105 °C for 3 h (method 930.15; [21]), and the ash content was subsequently determined after incineration at 550 °C for 2 h (method 942.05; [21]). The neutral and acid detergent fiber (NDF and ADF, respectively) analysis was performed with a fiber analyzer (Ankom Technology, Macedon, NY, USA) following the procedure of Van Soest et al. [18] without correction for residual ash. The ether extract (EE) and the N contents were respectively determined by the solvent extraction and by the Kjeldahl methods (methods 954.02 and 976.05, [21]).

The pH and the NH_3_ content of the inoculum were measured with a glass electrode pH meter (GLP 22, Crison Instruments, S.A. Barcelona, Spain) and an ammonia electrode (Ammonia Gas Sensing Combination Electrode, ©Hach Company, Loveland, CO, USA, 2001).

For the VFA analysis, the aliquots of the inoculum were centrifuged at 20,000 g for 30 min at 20 °C and the supernatant was then filtered using polypore 0.45 µm filters (Alltech Italia, Milan, Italy). The filtrate was injected into a high-performance liquid chromatography instrument (Perkin-Elmer, Norwalk, CN, USA), set to 220 nm according to the method described by Martillotti and Puppo [22].

#### 2.4.3. Microbial Analysis

Genomic DNA was extracted from about 700 µL of inoculum samples using the PowerSoil DNA extraction kit (MoBio Laboratories Inc., Carlsbad, CA, USA) with some modifications as described by Kong et al. [23] (2010). DNA concentration, eluted in 50 µL, was determined by NanoDrop One (Thermo Fisher Scientific Inc., Wilmington, DE, USA).

The quantitative polymerase chain reaction analysis was performed by CFX96 Real Time System (Bio-Rad Technologies Inc, Hercules, CA, USA) using iQ SYBR Green Supermix (Bio-Rad Technologies Inc, Hercules, CA, USA) mixed with 0.3 µL of each forward and reverse primer (0.3 µM), 8.4 µL of sterile water and 1 µL of gDNA to obtain a reaction volume of 20 µL. The amplification program included the denaturation step at 98 °C for 3 min followed by 40 cycles at 98 °C for 15 s, annealing at 60 °C for 30 s and elongation at 72 °C for 30 s.

To determine the specificity of the amplification of each primer, the melting curve was performed. The amplification efficiency was calculated using the formula: E = 10^(−1/slope)^ − 1. The relative abundance of the target bacterial groups or species was expressed in a proportion of the total bacteria 16S rRNA gene and was calculated using the following formula: 2^−ΔCT^.

#### 2.4.4. Statistical Analyses

The data from the in vitro tests (NDFd, RDP and GP, within each test and within each feed substrate) and the chemical and microbial composition of fermentation fluids differing for origin and preservation (pH values, VFA and ammonia contents and bacteria abundance) were statistically analysed as a factorial 2 × 4 completely randomised block (2 fermentation runs as blocks) design:

Y_ijk_= μ+ α_i_ + β_j_ + γ_k_+ (βγ)_jk_ + ε_ijk_,

where: y_ijk_ is the experimental data; μ is the overall mean; α_i_ is the random effect (block) of the fermentation run (i = 1, 2); β_j_ is the fixed effect of the type of inoculum (j = 1, 2); γ_k_ is the fixed effect of the inoculum preservation method (k = 1,4); and ε_ijk_ is the random error.

Within each in vitro test data, the Pearson correlation coefficient (r) was calculated for the 6 feeds tested in 2 runs with fermentation liquids, differing for origin (liquids from fermenter versus liquids from rumen, 12 data points) or for the type of conservation (W versus R, W versus C and W versus FD liquids, 12 data points for each correlation).

For all statistical analyses, the probability significance levels (*p*) were 0.05 and 0.01 (*p* < 0.05 and *p* ≤ 0.01, respectively).

## 3. Results

The chemical composition of feeds used as substrates for the in vitro tests and the characteristics of inocula are reported in Table 1 and Table 2, respectively.

Among the parameters reported in Table 2, the concentration of ammonia and the relative abundance of *Fibrobacter succinogenes* in fermentation fluids showed a significant interaction (*p* < 0.05) between the origin of the liquid and the preservation method. However, for both parameters the impact of the interaction in terms of contribution to the mean square of the model was much lower than that of the main effects as well the level of significance (*p* < 0.05 vs. *p* ≤ 0.01). Therefore, this study decided to show and to discuss the main effects of the model.

The liquid from the fermenter had a lower total VFA content, acetic percentage on total VFA, acetic:propionic ratio and ammonia concentration compared with the liquid from rumen (34.9 versus 122.1 mMol, 57.8 versus 63.3%, 2.9 versus 5.7 and 15.4 versus 26.1 mg/dL, respectively, *p* ≤ 0.01). On the contrary, the valeric and isovaleric acid percentages on total VFA were higher in fluid from the fermenter than the rumen liquid (1.8 versus 1.1% and 2.7 versus 1.4%, respectively, *p* ≤ 0.01). The FD preservation differed significantly (*p* ≤ 0.01) from the others for the lowest proportion of acetic, propionic and isovaleric acids and for the very high proportion of isobutyric acid. The FD also had a very low content of ammonia in comparison with W and R (6.4 versus 21.8–22.3 mg/dL, *p* ≤ 0.01), while the C liquid showed the highest concentration (32.6 mg/dL, *p* ≤ 0.01).

The relative abundances of the genus *Prevotella* and *Ruminococcus albus* group were lower (*p* < 0.01) in the liquid from the fermenter compared to the liquid from rumen, while the preservation method did not show effects. On the contrary, the *Fibrobacter succinogenes* was higher (*p* < 0.01) in the liquid from the fermenter and its relative abundance in W and R liquids was higher (*p* < 0.01) than the others.

The results of the in vitro tests on the six feeds and the correlation coefficients calculated on the six feeds by using the sets of two inocula differing for origin (liquid from rumen versus liquid from fermenter) or preservation (W versus R, W versus C and W versus FD liquids) are reported in Table 3 and Table 4, respectively. The in vitro tests used different inoculum and were performed in eight subsequent incubation sessions by each Lab. To correct any possible effect of the incubations session, a standard rumen fluid was included in each fermentation, which was used on the same substrate (corn silage) as the control. The data from the standard allowed the exclusion of any appreciable effect of the incubation session and the in vitro results from the different incubation sessions did not require corrections.

The NDFd data of the corn silage samples for the FD mode of preservation were removed from the analysis, having an abnormal fermentation due to technical problems.

The utilization of the liquid from the fermenter determined a significant NDFd reduction for three feeds (corn silage, wheat bran, and distillers) and the correlation analysis indicated a close relationship for NDFd between the liquid from rumen and diluted liquids from the fermenter (r = 0.960, *p* ≤ 0.01). Considering the preservation of the inoculum, the W and R liquids were not different for all the tested feeds and the correlation indicated a close correspondence (r = 0.985, *p* ≤ 0.01). The C and FD liquids depressed (*p* ≤ 0.01) the NDFd for two and three feeds, respectively. However, the correlation W versus C was high (r = 0.905, *p* ≤ 0.01), while the W versus FD was non-significant.

The RDP results indicated a good correspondence between fluid from the fermenter and liquid from the rumen liquids and the correlation between the liquid from rumen and the liquid from the fermenter data was high (r = 0.837, *p* ≤ 0.01). The R and C liquids gave similar RDP results to W liquid for all feeds and the regression W versus R showed a good correspondence of values (r = 0.892), while the W versus C was not significant. The RDP obtained with the FD liquid were quite variable, with the values for two feeds being higher than those obtained with W, while for the soya, the opposite was true, and the correlation W versus FD was not significant.

Unlike NDFd and RDP, the gas production showed a significant interaction—inoculum × preservation (*p* ≤ 0.01)—for some feeds (corn silage, wheat bran, and distillers). However, the impact of the interaction in terms of the contribution to the mean square of the model was much lower than that of the main effects as well the level of significance (*p* < 0.05 versus *p* ≤ 0.01). Therefore, it was decided to show the results and to discuss the main effects of the model. For all the feeds, the liquid from rumen gave higher gas production than the liquid from the fermenter (*p* ≤ 0.01 and, only for soya, *p* < 0.05).

Moreover, the preservation method had a statistical effect on the gas production (*p* ≤ 0.01 and, only for soya and barley, *p* < 0.05). In general, the gas production of C was not statistically different from W and R liquids, except for wheat bran and barley where C gas production was not statistically different from W. The FD liquid generated the lowest yields of gas and all the ingredients differed significantly from those of the W liquid. All correlations of preserved liquids with W were statistically significant (r = 0.797–0.921, *p* ≤ 0.01).

## 4. Discussion

### 4.1. Type of Inoculum

The present work used a rumen fluid collected from cows immediately after slaughter in controlled conditions (animals fed a known diet, not slaughtered in emergency, in good health status, transported from farms located near the slaughterhouse, rumen fluid sampled within 20 min of slaughter) as a reference in vivo rumen liquor. To our knowledge, there are no specific comparisons between rumen fluid collected via cannula, through the esophageal tube and at slaughtering. The authors reason that rumen fluid collected at slaughter in controlled conditions has a limited difference from that sampled by other methods. This is based on our previous work [24] where a close relationship between NDF degradability measured in vitro (average of rumen collected by different methods) and in situ was found. Our recent data [25] showed comparable values of in vitro NDF degradability using rumen fluids from slaughtered cows and from cows with rumen cannula fed similar diets. Moreover, the collection at slaughter is an accepted method of sampling rumen fluid for microbiota studies by the Rumen Microbial Genomics Network [26] and it is mentioned as an alternative to sampling via cannula [2].

A first aim of this study was to compare the fresh rumen inoculum with that obtained from a continuous culture system (RCF, see Figure 1). The data from the literature [4,5] showed that fermentation liquids from continuous cultures are less concentrated than rumen fluids collected directly from the rumen in terms of fermentation metabolites. This is confirmed also by data of this experiment, where a particularly low VFA concentration was found in the continuous fermenter liquids, being approximately 30% of the VFA concentration of rumen fluid. The concentration of VFAs measured in the rumen liquids were comparable to those found in the literature, while those of the RCF liquids were slightly lower than those obtained previously with the same in vitro system [14,27]. In addition, the ammonia reduction in the fermenter liquids was less marked than VFA, being approximately 60% of the ammonia in the rumen fluid. Moreover, a higher proportion of valeric and isovaleric acids was found in RCF liquids. These latter acids mainly originated from the metabolism of amino acids and this agrees with the relatively high ammonia concentration mentioned above.

The microbial population was represented in this study by only some bacterial strains and was affected by the type of inoculum. After the incubation period in the RCF, *Prevotella* genus and *Ruminococcus albus* group decreased the relative abundance, while *Fibrobacter succinogenes* increased, compared to the rumen liquids. *F. succinogenes,* both in the liquid from rumen and in the RCF liquids, was present at higher percentages than the *R. albus* group, as reported also from by Soto et al. [6,7] and by Koike et al. [28]. Our limited observations on the bacterial rumen microbiota confirm that the RCF in vitro environment shifts bacteria composition, because it depresses some strains and is favourable to others.

Despite the previously discussed differences between the two types of inoculum in terms of metabolites and microbiota concentration, the effects on the in vitro tests were less pronounced than expected, at least for NDF and crude protein degradability. On average, compared to the rumen fluid, the utilization of fermenter liquid inoculum determined a small reduction of NDFd (−12%), while the degradability of protein was slightly increased (+5%). Furthermore, the correspondence of NDFd data obtained on the six ingredients by the two inoculum was very close (r: 0.960, *p* ≤ 0.01). On the contrary, gas production was depressed by 20% with the usage of RCF, but also in this case, there was a close correlation between the values obtained with the two types of inoculum (r: 0.939, *p* ≤ 0.01). Therefore, despite the differences described above in metabolite and microbiota composition, the overall in vitro fermentability and the ranking of feeds in term of in vitro rumen fermentation do not seem to be greatly affected using the RCF fluid in comparison to rumen inoculum. This surprising result could be in part explained because the in vitro tests work at a high ratio of fermentation liquid to substrate (20, 50 and 180 mL/g of DM substrate for RDP, GP and NDFd, respectively). In such conditions, there is an abundance of degrading capacity and it could be speculated that the RCF fermenting fluid is not so diluted to greatly reduce the fermentability measured by the in vitro tests, at least for the fiber and protein fractions.

### 4.2. Method of Inoculum Preservation

A second aim of this study was to compare the fresh rumen inocula with those preserved by refrigeration, chilling and freeze-drying (Figure 1). The rumen fluid maintained at 39 °C for 5–6 h prior to incubation represented the reference rumen fluid because Robinson et al. [29] demonstrated that the storage in such conditions had no detrimental effects on in vitro NDF fermentation. The results of the present study show that rumen fluid refrigerated for 5–6 h had fermentative parameters very close to that of the fluid preserved at the animal body temperature. On the contrary, freezing at −80 °C determined a high ammonia increment and the freeze-drying process caused pronounced variations in comparison with fresh rumen fluid because both acetic and propionic acids proportions were reduced, and the iso-butyric increased abnormally and the ammonia was severely reduced.

The NDFd values obtained from the fluid preserved by refrigeration were very similar to those attained from the inoculum maintained at 39 °C, as previously found by Robinson et al. [29]. A similar situation was found also for the RDP values, apart from the two forage samples. The same close relationship between refrigerated and fluid from rumen was also the case for gas production and this result confirms those found by Hervás et al. [30], who suggested that the preservation of rumen fluid for up to 6 h in crushed ice is a practical alternative to the use of freshly collected rumen fluid as inoculum for in vitro ruminal fermentation studies. Therefore, refrigerated inoculum can be a more practical system of storing rumen inoculum during transport from the site of collection to the laboratory of utilization within a 4 to 6 h period.

The possibility of storing the inoculum for long periods was evaluated in terms of chilling at −80 °C and by freeze-drying. The chilled inoculum determined a limited reduction of NDF degradation (−14%) in comparison with the warm inoculum and there was a satisfactory relationship between feeds (r: 0.905).

Furthermore, the degradation of protein was, on average, not depressed and this agrees with the findings of Luchini et al. [31] who suggested that rumen inoculum preserved frozen might be used for in vitro rumen protein degradation experiments. However, the regression between feeds showed an insufficient degree of relation. 

Finally, less gas was produced by the chilled inocula (−20%) with a satisfactory relationship among feeds (r: 0.797). The protection of the microbiota from freezing damage can be obtained by adding organic chemicals with cryoprotectant properties but these compounds can interfere with the subsequent in vitro tests because they are fermentable substrates (e.g., glycerol, [12]) or can have toxic effects (e.g., dimethyl sulfoxide). A possible improvement of our chilling procedure [9,12] could be to avoid freezing large amounts of inoculum and to divide it into small amounts (e.g., 30–50 mL) because the high surface/volume accelerates the freezing.

The freeze-drying process produced the lowest fermentation activity and a very poor relation with fresh liquid among feeds, confirming similar findings obtained by Belanche et al. [9]. Several methodological factors can explain these poor results, such as the absence of cryoprotectants, the procedure of pellet preparation in terms of centrifugation intensity, and an improper process of rehydration. Systemic experimental work would be necessary to evaluate the role of specific conditions on the microbiota survival throughout whole process. Finally, it is worth noting that both preservation methods based on low temperatures, determined a drastic drop in the relative abundance of the *Fibrobacter succinogenes,* which plays a main role in fiber degradation. This variation could be associated with a depression of degradability and, consequently, with the low NDFD and GP values measured.

## 5. Conclusions

The results from the present trial indicate that fermentation liquid from rumen continuous fermenters can be used to generate inoculum for in vitro purposes. This result has relevant implications, not only in terms of artificially generating rumen fermenting fluids by RCF systems, but also from the perspective of the standardization of the fermentation liquids by adopting controlled fermentation conditions (e.g., substrate, dilution rate of saliva, fermentation, and pH). Short-term refrigeration is confirmed to be a valuable storage system which has practical advantages in managing rumen fluid, particularly in the case of laboratories distant from collection sites. Finally, low fermentative capacity was found for chilled and, particularly, for freeze-dried liquids, and the procedures adopted to obtain such preserved inoculum probably require substantial improvements.

## Figures and Tables

**Table 1:** Chemical composition of feeds used as substrates in the in vitro tests.

Feeds	DM	Ash	CP	EE	NDF	ADF
	%	% DM	% DM	% DM	% DM	% DM
Corn silage ^1^	91.88	4.16	7.67	3.10	36.79	20.74
Wheat bran	89.71	5.98	16.74	3.47	47.80	13.40
Meadow hay	95.54	11.30	7.34	1.45	58.38	34.82
Distillers	89.15	5.87	34.32	7.79	42.87	11.86
Soya meal, extr.	88.88	6.95	46.09	1.19	21.11	8.88
Barley	89.79	3.08	10.65	1.55	31.89	8.30

^1^ pre-dried samples at 60 °C.

**Table 2:** Volatile fatty acid (VFA) content, pH, ammonia (NH_3_) content and bacteria abundance of fermentation liquids from inoculum differing for origin and for conservation.

Items	Type of Inoculum (TI)		Type of Conservation ^1^ (TC)				TI ^2^	TC ^2^	TI × TC ^2^	RMSE ^2^
	From Rumen	From Fermenter	Warm (W)	Refrigerated (R)	Chilled (C)	Freeze—Dried (FD)				
Samples, *n*	8	8	4	4	4	4				
Total VFA (mMol)	122.1	34.9	76.4	74.1	100.3	63.4	**	ns	ns	25.98
VFA composition (% of total VFA)										
Acetic (A)	63.3	57.8	67.3 ^A^	66.0 ^A^	65.7 ^A^	43.4 ^B^	*	**	ns	3.72
Propionic (P)	12.5	20.2	18.2 ^A^	18.5 ^A^	16.9 ^A^	11.9 ^B^	**	**	ns	1.84
Isobutyric	10.9	9.6	0.4 ^B^	0.4 ^B^	4.2 ^B^	36.0 ^A^	ns	**	ns	5.77
Butyric	10.5	8.0	10.1	10.8	9.5	6.5	ns	ns	ns	2.35
Isovaleric	1.4	2.7	2.1 ^A^	2.4 ^A^	2.3 ^A^	1.2 ^B^	**	**	ns	0.34
Valeric	1.1	1.8	1.6	1.6	1.5	1.2	*	ns	ns	0.40
A:P	5.7	2.9	3.9	3.8	4.0	5.4	**	ns	ns	1.27
pH	6.4	6.7	6.5	6.5	6.5	6.8	ns	ns	ns	0.51
NH_3_ (mg/dL)	26.1	15.4	21.8 ^B^	22.3 ^B^	32.6 ^A^	6.4 ^C^	**	**	*	3.28
Relative abundance (% of total bacteria)										
Genus *Prevotella*	45.1	19.7	25.8	31.7	34.6	37.5	**	ns	ns	8.90
*Fibrobacter succinogenes*	0.39	2.04	2.36 ^A^	2.10 ^A^	0.39 ^B^	0.01 ^B^	**	**	*	0.63
*Ruminococcus albus* group	0.017	0.003	0.013	0.010	0.011	0.006	**	ns	ns	0.0056

^1^ Means of the type of conservation with different superscripts (A, B, C) are statistically different. ^2,^*: *p* < 0.05; **: *p* ≤ 0.01; ns: not significant; RMSE: root mean square error.

**Table 3:** In vitro degradability of NDF (NDFd), CP (RDP), and gas production (at 24 h of fermentation, GP) of six substrates using rumen inoculum differing for origin and for conservation method.

Items	Type of Inoculum (TI)		Type of Conservation ^1^ (TC)				TI ^2^	TC ^2^	TI × TC ^2^	RMSE ^2^
	From Rumen	From Fermenter	Warm (W)	Refrigerated (R)	Chilled (C)	Freeze-Dried (FD)				
NDFd (%)										
Corn silage	37.5	30.1	39.0 ^A^	40.0 ^A^	22.5 ^B^	-	*	**	ns	6.34
Wheat bran	45.9	38.2	46.7 ^A^	46.2 ^A^	39.3 ^B^	36.0 ^B^	**	**	ns	3.40
Meadow hay	44.5	40.7	51.8 ^A^	47.9 ^A,B^	39.0 ^B,C^	31.8 ^C^	ns	**	ns	5.36
Distillers	52.5	41.3	49.4	51.7	47.9	38.5	*	ns	ns	6.85
Soya meal, extr.	81.4	78.5	92.2 ^A^	93.7 ^A^	85.5 ^A^	48.5 ^B^	ns	**	ns	6.26
Barley	59.4	54.3	58.0	58.8	55.7	55.1	ns	ns	ns	5.39
RDP (%)										
Corn silage	66.8	66.2	57.0 ^b^	49.8 ^b^	66.0 ^b^	93.3 ^a^	ns	*	ns	15.21
Wheat bran	59.9	61.9	58.1	58.9	60.4	66.2	ns	ns	ns	10.20
Meadow hay	39.0	45.6	32.5 ^b^	27.8 ^b^	44.3 ^b^	64.7 ^a^	ns	*	ns	11.35
Distillers	33.8	34.5	34.8	37.5	33.8	30.6	ns	ns	ns	5.00
Soya meal, extr.	45.1	53.4	52.8 ^A^	56.8 ^A^	47.8 ^A,B^	39.7 ^B^	*	*	ns	5.10
Barley	59.0	57.4	51.8	49.7	60.4	71.0	ns	ns	ns	17.38
GP (mL)										
Corn silage	223	193	242 ^A^	234 ^A^	184 ^B^	172 ^B^	**	**	**	26.13
Wheat bran	183	146	176 ^A,B^	188 ^A^	157 ^B,C^	136 ^C^	**	**	**	18.40
Meadow hay	162	119	177 ^A^	198 ^A^	124 ^B^	64 ^C^	**	**	ns	24.81
Distillers	193	153	197 ^A^	207 ^A^	156 ^B^	131 ^B^	**	**	**	50.44
Soya meal, extr.	178	134	176 ^B^	213 ^A^	136 ^C^	100 ^D^	*	*	ns	23.31
Barley	266	226	263 ^A,B^	264 ^A^	231 ^A,B^	226 ^B^	**	*	ns	27.85

^1^ Means of the type of conservation with different superscripts (A, B, C and a, b) are statistically different. ^2^^,^*: *p* < 0.05; **: *p* ≤ 0.01; ns: not significant; RMSE: root mean square error.

**Table 4:** Pearson correlation coefficients (r) of in vitro data obtained for the six feeds used as substrates in two fermentation run (12 observations) by using inocula differing for origin and for conservation.

	Type of Inoculum (TI)		Type of Conservation (TC)					
	Rumen vs. Fermenter		Warm vs. Refrigerated		Warm vs. Chilled		Warm vs. Freeze-Dried	
	r	*p* ^1^	r	*p* ^1^	r	*p* ^1^	r	*p* ^1^
NDFd ^2^	0.960	**	0.985	**	0.905	**	0.554	ns
RDP ^3^	0.837	**	0.892	**	0.345	ns	0.431	ns
GP ^4^	0.939	**	0.921	**	0.797	**	0.850	**

^1^ statistical significance of r: **: *p* ≤ 0.01; ns: not significant. ^2^ NDFd: NDF degradability; ^3^ RDP: CP degradability; ^4^ GP: gas production (at 24 h of fermentation).
